# Efficacy and safety of anti-obesity herbal medicine focused on pattern identification: A systematic review and meta-analysis

**DOI:** 10.1097/MD.0000000000032087

**Published:** 2022-12-16

**Authors:** Seohyun Park, Dongho Keum, Hojun Kim

**Affiliations:** a Department of Rehabilitation Medicine of Korean Medicine, Dongguk University Bundang Hospital, Seongnam-si, Gyeonggi-do, Republic of Korea; b Department of Rehabilitation Medicine of Korean Medicine, Dongguk University Ilsan Hospital, Ilsan-si, Gyeonggi-do, Republic of Korea.

**Keywords:** herbal medicine, meta-analysis, obesity, pattern identification, systematic review

## Abstract

Herbal medicine based on pattern identification (PI) is used widely in Traditional Chinese Medicine. Proper herbal medicine based on PI has been suggested for effective weight reduction and decreasing the adverse events. This systematic review examined the effectiveness and safety of herbal medicine, focusing on PI in treating obesity.

**Methods:**

Eight electric databases were used for searching randomized controlled trials (RCT) (to August 31, 2021). RCTs which prescribed herbal medicine to obese patients based on PI were included. Body weight (BW) and body mass index (BMI) were the primary outcomes. The risk of bias was assessed using Cochrane risk of bias tool, and the meta-analysis was conducted. Grading the evidence was conducted by using GRADEpro.

**Results:**

Sixteen RCTs (1052 patients) were included: 2 studies compared herbal medicine to placebo (128 patients); 2 studies compared them to western medication (161 patients); 12 studies compared them with usual care (763 patients). The meta-analysis showed that the herbal formulas reduced the BW and BMI without significant Adverse events compared to the control group (BW: mean difference  = –4.10, 95% confidence interval: –5.14 to –3.06, *I*^2^ = 2% and BMI: mean difference = –1.53, 95% confidence interval: –1.88 to –1.19, *I*^2^ = 25%). Moderate-quality evidence on the primary outcomes was found.

**Conclusions:**

Herbal medicine – has good clinical efficacy and safety in treating obesity. This study has limitations that some literatures with high risk of bias in blinding or without using a standardized diagnosis of PI were included. However, the current evidence suggests the possibility of precision medicine using PI.

## 1. Introduction

Obesity is defined as excessive fat accumulation with health risks.^[1–3]^ Worldwide, 39% of adults were estimated to be obese in 2016, and the number has increased constantly in recent years.^[4,5]^ Obesity-related health consequences follow the growth in the prevalence of obesity because it is a major risk factor for non-communicable diseases, such as cardiovascular disease, diabetes, musculoskeletal disorder, and even some cancers.^[4–6]^ In Traditional Chinese Medicine (TCM), obesity is caused by dampness, phlegm, blood stasis, heat accumulation in the stomach and spleen, *qi* deficiency, spleen deficiency, and *yang* deficiency.^[1–3]^ Hence, treatments are always recommended according to the patient’s unique physical and environmental factors.^[1,3]^

Herbal medicine is a common intervention for obesity in TCM and is recommended based on individual characteristics using pattern identification (PI). PI is a characteristic point in the diagnosis and treatment of diseases. Even with the same disease, the pathogenic mechanism can be different, which means that the treatment should also be different. Various symptoms have been identified and categorized to determine the pathogenesis and mechanism of the disease, which is called pattern identification. This PI was applied to treatment, which enables more precise methods for treatment. In Korea, the 6 PI types, developed by the Korea institute of oriental medicine, are used widely in treating obesity: phlegm, food retention, blood stasis, liver-*qi* stagnation, deficiency of *yang*, and deficiency of spleen. Previous reports have suggested that herbal medicine based on PI can be an effective and safe approach to improving weight reduction.^[7–12]^ Many attempts have been made to determine differences in the characteristics of obese patients according to PI.^[7–12]^ Furthermore, it was reported that herbal medicine with an unmatched PI could lead to more frequent adverse events (AE), separate from its weight loss effect.^[12]^

In recent years, there has been increasing interest in precision medicine in various study fields. PI can be an attractive option for individualized medicine because PI has been used to categorize patients and select proper treatment according to their symptoms, pathogenesis, and treatment responses. The association between PI and precision medicine has been reported, and the possibility of PI as a precision medicine for diseases other than obesity is being actively studied.^[13–16]^ The efficacy and safety of acupuncture using PI for sleep disorders were reported, and the treatment for the common cold and COVID-19 using PI were studied.^[14,15]^ Many studies suggested the possibility of PI as individualization in modern medicine; it can contribute to clinical research and pharmacological research.^[17,18]^ PI help the clinical trial design in that researchers are able to expect responsive or non-responsive case using PI, so that the most appropriate patients for the intervention can be chosen.^[18]^ Furthermore, PI allow us to anticipate the results of applying renew drug to patients with specific type of PI by comparing the results of applying similar old drugs to specific patients with the same type of PI.^[18]^

In the present situation, few studies focused on PI have been published since the early 2010s.^[7–11]^ Most studies that reviewed the effectiveness of herbal medicine on obesity did not focus on PI or its efficacy.^[17–24]^ The mechanism or specific points that are meaningful to obesity treatment, such as appetite or adipose tissue growth, were only reported from those articles.^[19–24]^; The effectiveness and safety of several single herbs were reported, but they were limited to the several selected medical plant or review on studies whose subjects were not human.^[19–23]^ It was also reported that *mahuang* and ephedrine in the appropriate dose were effective in reducing the weight safely compared to the control group.^[24]^ On the other hand, those studies reviewed just single herbs and did not cover common types of herbal medicine consisting of various herbs.^[24]^ Few reports studied herbal medicine that consisted of various herbs based on TCM theory, but those studies also had limitations.^[25,26]^ After a systematic review and meta-analysis, it was reported that *bangpoongtongsung-san (BTS*) and *taeeumjowi-tang (TEJWT*) showed positive results in body weight (BW), body mass index (BMI), and waist circumference (WC) without severe AEs, but that focused on just 1 selected herbal formula.^[25]^ The review of herbal medicine containing several herbs was also reported, but it did not conduct an additional meta-analysis, so the effect size of herbal medicine could not be estimated.^[26]^There is difficulty in clarifying whether herbal medicine based on PI is more effective and safer, because the lack of number of studies designed according to the PI. However, it is meaningful to focus on PI and evaluate the efficacy and safety of herbal medicine, considering that PI is the most basic and widely used method for determining the direction of treatment in obesity.

Therefore, this study reviewed randomized controlled trials (RCTs) related to herbal medicine based on PI and attempted to find the characteristics of PI and results of herbal medicine with PI. This study evaluated the effectiveness and safety of herbal medicine in obesity with a focus on PI.

## 2. Methods

This review was conducted according to the Preferred Reporting Items for Systematic Reviews and Meta-Analyses (PRISMA) guidelines. The protocol of this study was registered on PROSPERO (CRD42021271425 Available from: https://www.crd.york.ac.uk/prospero/display_record.php?RecordID=271425).

### 2.1. Electronic searches and search strategy

The search was performed in August 2021 in the following eight electronic databases: PubMed (MEDLINE), Cochrane Central Register of Controlled Trials, EMBASE, China National Knowledge Infrastructure (CNKI), CiNii, KoreaMed, Science-on, and Oriental Medicine Advanced Searching Integrated System (OASIS). The following terms were searched: herbal medicine (including a Mesh search using “obes*” OR “weight gain*” OR “weight loss” OR “body mass ind*” OR “adipos*” OR “overweight” OR “weight reduc*” OR “weight loss” OR “weight maint*” OR “weight decreas*” OR “weight control*”) and obesity (including a Mesh search using “herbal medicine” OR “plants, medicinal” OR “medicine, traditional” OR “drugs, Chinese herbal” Or “medicine, Korean traditional” OR “medicine, Kampo” OR “traditional Chinese medicine” OR “plant extracts”). Supplement 1 reveals the specific search terms for each database. The searches were conducted with each electronic database’s supported language, and there was no language restriction in the search strategy.

After the literature search, all duplicated articles were excluded. The titles and abstracts of all studies were examined, and irrelevant articles were excluded. Finally, the full-text articles were reviewed for relevant RCTs. To increase the sensitivity, “pattern identification” was not included in the search terms. All studies using PI were included after the first screening by the researcher.

### 2.2. Eligibility criteria and study selection

The titles and abstracts of the articles were examined, and only potentially relevant studies were selected. Only studies that recruited participants according to selected PI in advance, before the intervention was applied, were included.

#### 2.2.1. Types of studies.

RCTs with parallel-group designs were included. Non-RCTs, including mechanism studies, non-controlled studies, case reports, feasibility studies, and reviews, were also excluded.

#### 2.2.2. Types of participants.

Obese participants with a BMI over 25 kg/m^2^ were included. Participants under 18 years were excluded. Participants with complications or secondary obesity were also excluded.

#### 2.2.3. Types of intervention.

RCTs that examined the effects of herbal medicine based on PI were included. Only herbal medicine, a prescription based on the theory of TCM, was included. Herbal medications without a proper prescription or just a mixture of several herbs without TCM theory were excluded. There were no limits on the forms of herbal medicine, such as decoction, capsule, tablet, pill, powder, and extracts. Studies involving herbal medicine combined with other therapies as an experimental intervention were excluded. Herbal medicines with lifestyle changes, including dietary modification or exercise modification, were included if the modifications were applied to both groups. The control interventions included a placebo, usual care, other medication, and managing dietary or exercise habits. Other medications indicated western medication, known as oral medication, such as orlistat or liraglutide, but it did not include operative methods. Usual care means management of lifestyle, including dietary modification or increases in physical activity.

#### 2.2.4. Types of outcome measurements.

The primary outcomes were BW and BMI. Additional outcomes were WC, hip circumference (HC), waist-hip ratio (WHR), and AEs.

### 2.3. Data extraction

The following data were extracted by 2 reviewers (SHP, DHK): study design, sample size, characteristics of participants, intervention, comparators, treatment duration, outcome measurements, AE, and information for an assessment of the study quality. Missing data or queries were followed-up with the original authors via email, if needed.

### 2.4. Assessment of risk of bias

The risk of bias assessment was performed using the “risk of bias” tool from Cochrane Collaboration. The tool consisted of 7 domains: sequence generation, allocation concealment, blinding of participants and personnel, blinding of outcome assessors, incomplete outcome data, selective outcome reporting, and other bias. The risk of bias for each domain was rated as “low risk, “high risk,” or “unclear risk.” Two reviewers (SHP and DHK) assessed the risk of bias independently. When there were any disagreements or discrepancies, a third reviewer (HK) made the final decision.

### 2.5. Summary measures and synthesis of results

The Review Manager software for Windows (RevMan ver.5.3.; Copenhagen; The Nordic Cochrane Center, The Cochrane Collaboration, 2014) was used for data synthesis. The meta-analysis and the evaluation of risk ratio (RR) or standard mean difference (SMD) were performed. A random effect model with 95% confidence was selected to calculate the pooled effect size estimates. The *I*-squared test was used to evaluated the heterogeneity. Heterogeneity was considered based on a rough guide to interpretation as follows: an *I*^2^ value from 0% to 40% might not be important; an *I*^2^ value from 30% to 60% may represent moderate heterogeneity; an *I*^2^ value from 50% to 90% may indicate substantial heterogeneity; an *I*^2^ value from 75% to 100% means considerable heterogeneity.

Considering the heterogeneity, subgroup analysis was conducted according to the different comparisons. Furthermore, funnel plots were used to assess the publication bias when there were more than 10 identified studies in the meta-analysis.

### 2.6. Assessment of quality of evidence for each outcome

The GRADEpro Guideline Development Tool (https://community.cochrane.org/help/tools-and-software/gradepro-gdt, version3.6.) was used to assess the quality of evidence for each outcome across the studies. A “Summary of findings” tables was generated using GRADEpro GDO software (GRADEpro GDT, available at https://www.gradepro.org), and the tables were imported into the review.

The quality of evidence was described as “high,” “moderate,” “low,” or “very low,” using the GRADE framework and applied to all primary and additional outcomes. The risk of bias, indirectness, inconsistency, imprecision, and publication bias for all studies was estimated. The quality of evidence started high because only randomized controlled studies were included. The quality of evidence was downgraded according to the above domains: it was downgraded by 1 point when the domain was estimated to be serious and 2 points when it was assessed to be very serious.

In relation to the risk of bias, not serious meant there was no risk of bias in more than 80% of the studies included or studies with large sample sizes. It was considered serious if there was a high risk of blinding. If most studies had a high risk of bias, it was evaluated as very serious.

In relation to the inconsistency, not serious meant that the *I*^2^ value was less than 50%, or the results of studies had the same direction even if the *I*^2^ value was more than 50%. If the *I*^2^ value ranged from 50% to 75% or the results of the studies had the same direction, even if the *I*^2^ value was over 75%, it was estimated to be serious. An *I*^2^ value over 75% meant very serious.

In relation to the indirection, serious or very serious were determined based on the outcome measurement. If the study presented outcomes unrelated to obesity, such as mineral components, it was estimated to be very serious. If there was only an effective rate resulting from anti-obesity, it was assessed to be serious.

In relation to imprecision, a total sample size of more than 400 was not estimated to be serious. A total sample size less than 400 or insignificant result was assessed to be serious with or without a sample size over 400.

In other considerations, publication bias was considered serious. Redundant publications, a publication of 2 or more articles derived from a single study, and conflict of interest with a sponsor were estimated to be serious.

This study evaluated the importance of outcomes as follows: BW and BMI were critical with 9 points; WC, HC, and WHR were critical with 8 points; AE was critical with 7 points.

## 3. Results

### 3.1. Study inclusion

**Figure 1.**
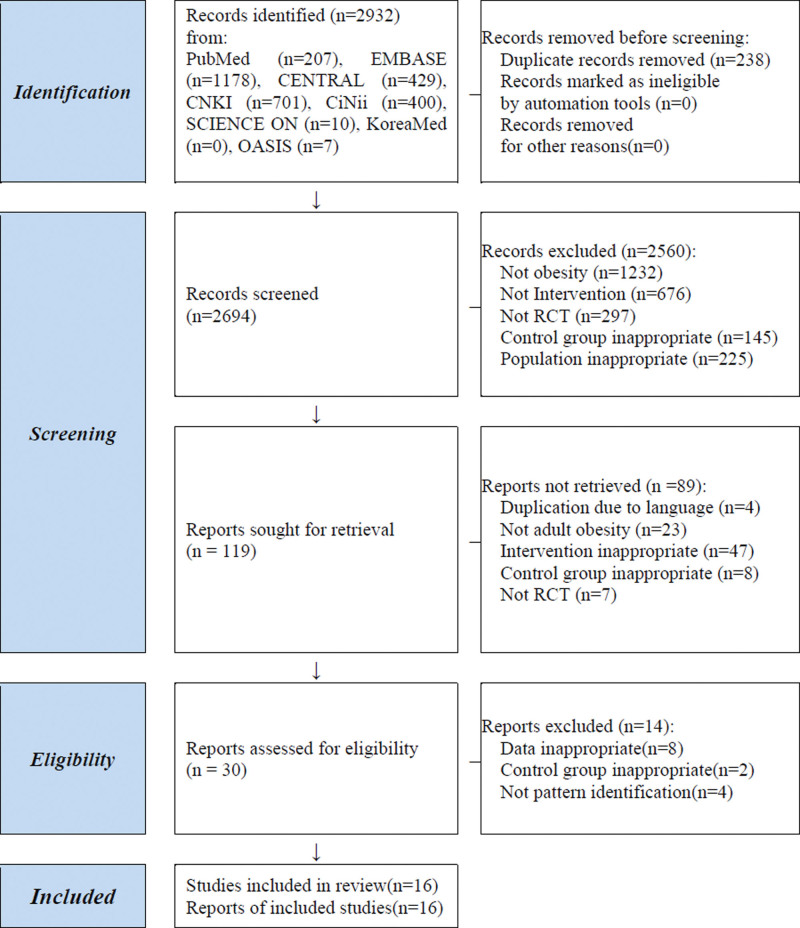
Flow chart of the study selection process. RCT = randomized controlled trial.

A total of 2932 citations were identified. Studies that did not use PI or inappropriate subjects, comparators, or data were excluded. Finally, 16 RCTs were included (1052 patients). According to the comparison, 2 RCTs (128 patients) compared herbal medicine to a placebo; 2 RCTs (161 patients) compared them to Western medication; 12 RCTs (763 patients) compared them to usual care, including modulation of the diet or exercise (Fig.1).

### 3.2. Study characteristics

Table 1 lists the characteristics of the included studies (16 RCTs). All studies included were performed in China. The number of patients involved in the studies ranged from 25 to 40 in the treatment or control groups. In total, 14 kinds of herbal formulas were involved. The forms of the intervention included decoction (12 RCTs), capsule or tablet (4 RCTs), and powder (1 RCT). Two RCTs used a placebo as a comparator, and 2 RCTs used orlistat for comparison. Twelve RCTs used diet and exercise control as a comparator of usual care. Most included reasonable diets or limited calories and aerobic exercise. The treatment duration ranged from 28 days to 3 months; 12 weeks (3 months) was mostly used (8 RCTs), followed by 8 weeks (2 months or 60 days) (6 RCTs). Modulation of diet and exercise was used in 15 RCTs. Only 2 studies used both BMI and BW as the outcome measure. Nine, 6, and 7 RCTs used WC, HC, and WHR, respectively, as an outcome measure. Eight RCTs reported the result of AE, and most were not significant in either group.

**Table 1:** Characteristics of studies.

Author (Year) Country	Intervention	Subjects	Treatment duration	Outcome measurements	Main results		Dietary modification	Effective variables	Adverse events
	Control						Exercise modification		
Herbal medicine vs placebo									
Xu H.^[27]^ (2008) China	Fat and gas capsules (n = 30)	Simple obesity (BMI > 28 kg/m^2^)	12 weeks	Tanshi symptom score, BW, BMI, WC, HC, F%, TG, TC, HDL-C, LDL-C, FPG, blood uric acid, effective rate(slimming effect, Tanshi symptom improvement)	1. BW 2. BMI 3. WC 4. HC	1. −7.60 ± 11.80 vs −2.90 ± 13.37 2. −4.10 ± 2.88 vs −1.10 ± 3.96 3. −6.00 ± 8.88 vs −1.40 ± 10.93 4. −7.30 ± 14.17 vs −4.70 ± 10.65	Reasonable diet	Tanshi symptom score, WC, BW, BMI, F%, HDL-C, LDL-C, blood uric acid, effective rate(slimming effect, Tanshi symptom improvement)	There were no significant adverse events.
	Placebo (n = 30)						Moderate exercise structure-based treatment		
Sheng et al^[28]^ (2017) China	*JianPi ShuGan* Formula (n = 34)	Simple obesity (BMI > 28 kg/m^2^)	28 days	Effective rate, BMI, TCM score, TG, TC, HDL-C, LDL-C, Leptin, Adiponectin	1. BMI	1. −1.77 ± 2.90 vs −0.23 ± 3.91	-	Effective rate, BMI, TCM score, TG, TC, HDL-C, LDL-C, Leptin, Adiponectin	There were no significant adverse events.
	Placebo (n = 34)						-		
Herbal medicine vs western-medication									
Hou et al^[29]^ (2019) China	*Xiere Huazhuo* decoction (n = 41)	Simple obesity (BMI > 28 kg/m^2^)	12 weeks	BW, BMI, FM, WC, WHR, TFM, TFM%, TC, TG, LDL, HDL, FPG, PPG, LEP, ADP, IL-6, TNF-α	1. BW 2. BMI 3. WC 4. WHR	1. −2.6 ± 3.8 vs −1.6 ± 2.7 2. −2.09 ± 1.4 vs −0.7 ± 1.0 3. −3.0 ± 3.8 vs −1.3 ± 3.8 4. −0.007 ± 0.006 vs −0.008 ± 0.008	1500 kcal/day or 1800 kcal/day	BW, BMI, FM, WC, TFM, LEP, TNF-α	Not reported
	Orlistat (n = 40)						40–60 min/once Over 5 times/week		
Wu et al^[30]^ (2020) China	*Liujunzi Tang* (n = 40)	Simple obesity (BMI > 25 kg/m^2^)	12 weeks	Effective rate, syndrome score, BW, BMI, FAT, Leptin, ADPN	1. BW 2. BMI	1. −7.93 ± 8.11 vs −3.93 ± 8.96 2. −2.40 ± 3.34 vs −1.39 ± 3.31	Balanced diet	Effective rate, syndrome score, BW, BMI, FAT, Leptin, ADPN	Not reported
	Orlistat (n = 40)						40 min/day		
Herbal medicine vs Usual care									
Feng et al^[31]^ (2015) China	*Wuling* powder (n = 33)	Simple obesity (BMI > 25 kg/m^2^)	8 weeks	BW, BMI, WC, HC, TC, LDL, HDL, TG, Effective rate	1. BW 2. BMI 3. WC 4. HC	1. −6.84 ± 12.14 vs −2.34 ± 12.26 2. −4.50 ± 5.08 vs −1.17 ± 5.13 3. −7.08 ± 13.11 vs −1.89 ± 13.30 4. −7.31 ± 13.45 vs −0.52 ± 13.64	Diet prescription	BW, BMI, WC, HC, TG, Effective rate	Not reported
	Diet exercise prescription (n = 32)						Exercise prescription (including aerobic exercise)		
Huang et al^[32]^ (2017) China	*Linggui Zhugan* decoction (n = 36)	Simple obesity (BMI > 28 kg/m^2^)	8 weeks	BW, BMI, TC, TG, LDL-C, HDL-C, Effective rate	1. BW 2. BMI	1. −5.43 ± 6.13 vs −3.09 ± 5.25 2. −3.26 ± 3.14 vs −2.25 ± 3.52	Reasonable diet	Effective rate, BW, BMI, TC, TG, LDL-C, HDL-C	There were no significant adverse events.
	Healthier diet and exercise (n = 36)						aerobic exercise Over 30 min/once Over 5 times/week		
Mai X.^[33]^ (2008) China	*Wu Lin* decoction (n = 30)	Simple obesity (BMI > 25 kg/m^2^)	8 weeks	Effective rate, BW, BMI, WC, TC, LDL-C, HDL-C, TG, FBG	1. BW 2. BMI 3. WC	1. −5.04 ± 2.17 vs −2.48 ± 2.28 2. −4.12 ± 0.27 vs −2.49 ± 0.48 3. −4.12 ± 0.27 vs −2.49 ± 0.48	5020–6275 kJ/day according to weight and height, balanced diet, drinking limitation	Effective rate, BW, BMI, WC, TG	e.g.: 2 (mild diarrhea) CG: 0
	Food and drink reducing weight to move prescription (n = 30)						aerobic exercise Over 30 min/once Over 3 times/week		
Wang et al^[34]^ (2019) China	*Sanziyangqin* decoction combined with *Yueju Pill* (n = 30) Diet and exericese (n = 30)	Simple obesity (BMI > 28 kg/m^2^)	60 days	BW, BMI, WC, HC, TC, LDL, TG, Effective rate, FBG	1. BW 2. BMI 3. WC 4. HC	1. −6.57 ± 12.09 vs −3.83 ± 10.91 2. −2.19 ± 1.66 vs −1.31 ± .80 3. −4.33 ± 8.41 vs −2.62 ± 9.44 4. −2.76 ± 7.54 vs −1.60 ± 7.83	Controlled diet according to height, weight, intensity of daily activity	Effective rate, BW, WC, HC, BMI, TC, TG	Not reported
							appropriate aerobic exercise - over 150 min/week (over 30 min/once) muscle resisted training – 3–5 times/weeks		
Wu W.^[35]^ (2016) China	*Bangpungtongsung-san* (n = 36)	Simple obesity (BMI > 28 kg/m^2^)	3 months	BW, BMI, WHR, TC, LDL-C, TG, Effective rate, TCM score, LP, APN	1. BW 2. BMI 3. WHR	1. −10.45 ± 9.89 vs −3.62 ± 9.04 2. −3.65 ± 1.96 vs −1.20 ± 2.09 3. −0.07 ± 0.05 vs −0.01 ± 0.03	1000–1200 kcal/day (Female) 1200–1600 kcal/day (Male)	Effective rate(TCM score, anti-obesity effect), BW, BMI, WHR, TC, TG, LP, APN	There were no significant adverse events.
	Basic treatment (diet control, appropriate aerobic exercise, health education) (n = 36)						Moderate aerobic exercise		
Xiao T.^[36]^ (2018) China	*Ze Xie Jia Wei* decoction (n = 30)	Simple obesity (BMI > 28 kg/m^2^)	1 month	BW, BMI, WC F%, TCM score, Effective rate	1. BW 2. BMI 3. WC	1. −2.01 ± 9.09 vs −1.87 ± 7.70 2. −0.79 ± 2.41 vs −0.70 ± 3.37 3. −2.79 ± 7.58 vs −3.25 ± 7.05	Controlled diet (reducing 500 kcal/day)	Effective rate, TCM score, BW, BMI, F%, WC	There were no significant adverse events.
	Basic treatment (n = 30)						appropriate aerobic exercise over 150 min/week (over 30 min/day) several times/weeks		
Zhu Y.^[37]^ (2017) China	*Dachaihu* decoction (n = 40)	Simple obesity (BMI > 28 kg/m^2^)	12 weeks	BW, BMI, WC, HC, WHR, WBC, RBC, PLT, ALT, AST, SCr, BUN, UA, TC, TG, LDL-C, HDL-C, Effective rate	1. BW 2. BMI 3. WC 4. HC 5. WHR	1. −11.31 ± 8.48 vs −0.66 ± 9.47 2. −4.13 ± 2.26 vs −0.23 ± 1.54 3. −4.37 ± 1.01 vs −1.31 ± 1.15 4. −3.78 ± 1.85 vs −1.12 ± 1.10 5. −0.01 ± 0.21 vs −0.01 ± 0.02	Appropriate diet	BW, BMI, WC, HC, TC, TG, LDL-C, HDL-C, Effective rate	There were no significant adverse events.
	Basic treatment (n = 35)						Appropriate exercise		
Li et al^[38]^ (2007) China	*Jianfei Tiaozhi capsule*(JTC) (n = 25)	Simple obesity (BMI > 28 kg/m^2^)	60 days	BW, BMI, F%, WC, HC, WHR, FBG, TG, INS, Leptin	1. BW 2. BMI 3. WC 4. HC 5. WHR	1. −6.18 ± 8.02 vs −3.00 ± 9.22 2. −2.30 ± .38 vs −1.01 ± 2.15 3. −3.10 ± .78 vs −1.30 ± 3.56 4. −2.40 ± 2.46 vs −1.08 ± 2.51 5. −0.01 ± 0.04 vs 0.00 ± 0.06	Diet control for reducing body weight	BW, BMI, F%, WC, HC, WHR, FBG, INS, Leptin	e.g.: 2 (mild diarrhea) CG: 0
	Basic treatment (n = 25)						appropriate aerobic exercise 30 min/once 3 times/weeks		
Li J.^[39]^ (2015) China	*Peilianmahuangfang* (n = 30)	Simple obesity (BMI > 28 kg/m^2^)	12 weeks	Effective rate, TCM score, BW, BMI, WHR, TC, TG, LDL-C, FPG, INS, IAPP, GLP-1	1. BW 2. BMI 3. WHR	1. −10.47 ± 10.55 vs −4.63 ± 8.23 2. −2.60 ± 2.64 vs −1.58 ± 2.63 3. −0.09 ± 0.07 vs −0.03 ± 0.05	1000–1200 kcal/day(Female) 1200–1600 kcal/day(Male)	Effective rate, TCM score, BW, BMI, WHR, TG, INS, IAPP, GLP-1	Not reported
	Basic treatment (health education, appropriate exercise, diet control, behavior intervention) (n = 30)						Moderate aerobic exercise		
Si et al^[40]^ (2014) China	*Wenshenjianpihuatan* decoction (n = 30)	Simple obesity (BMI > 28 kg/m^2^)	2 months	Effective rate, BW, BMI, WC, HC, WHR, TC, TG, LDL-C, HDL-C	1. BW 2. BMI 3. WC 4. HC 5. WHR	1. −6.86 ± 4.34 vs −0.96 ± 5.95 2. −5.84 ± .82 vs −3.40 ± 1.58 3. −5.69 ± 2.95 vs −3.34 ± 3.15 4. −5.64 ± 3.33 vs −3.64 ± 3.19 5. −0.01 ± 0.22 vs −0.01 ± 0.08	Appropriate diet	Effective rate, WC, BMI, WC, HC, WHR, TC, TG, LDL-C, HDL-C	Not reported
	Basic treatment (n = 30)						Appropriate exercise		
Yang Y.^[41]^ (2016) China	*Qutan Qingwei* decoction (n = 40)	Simple obesity (BMI > 28 kg/m^2^)	12 weeks	Effective rate, BW, F%, TG, TC, HDL-C	1. BW	1. −8.68 ± 7.71 vs −5.66 ± 6.81	Reasonable diet	Effective rate, BW, Fat rate, TG, TC, HDL-C	Not reported
	Basic treatment (n = 40)						appropriate aerobic exercise over 30 min/once over 3 times/weeks		
Yu H.^[42]^ (2016) China	*Peilianmahuangfang* (n = 31)	Simple obesity (BMI > 28 kg/m^2^)	12 weeks	BW, BMI, WHR, TG, TC, LDL-C, Effective rate, TCM score, LPS	1. BW 2. BMI 3. WHR	1. −9.70 ± 11.44 vs −5.67 ± 11.28 2. −3.40 ± 1.71 vs −1.97 ± 1.86 3. −0.10 ± 0.05 vs −0.03 ± 0.05	1000–1200 kcal/day (Female) 1200–1600 kcal/day (Male)	BW, BMI, WHR, TG, Effective rate, TCM score, LPS	Not reported
	Basic treatment (diet, exercise, behavior intervention, health education (n = 31)						45 min–1 hr/once 3-5 times/weeks		

BMI = body mass index, BW = body weight, CG = control group, EG = experimental group, FBG = fasting blood glucose, FPG = fasting plasma glucose, HC = hip circumference, HDL-C = High density lipoprotein, INS = insulin, F%; body fat percent, LDL-C = Low density lipoprotein, TC = total cholesterol, TCM = traditional Chinese medicine, TG = triglyceride, WC = waist circumference, WHR = waist-hip ratio

### 3.3. Pattern identification

Table 2 lists the classification of PI and the diagnostic criteria used for PI.

**Table 2:** Characteristics of pattern identification.

Pattern identification			The diagnostic criteria used for pattern identification	Reference
Major type	Subtype	Herbal medication		
Phlegm-dampness type	Phlegm-dampness with spleen deficiency	*Linggui Zhugan Decoction*	Diagnosis and efficacy evaluation criteria of simple obesity	^[32]^
		*Liujunzi Tang*	Diagnosis and efficacy evaluation criteria of simple obesity	^[30]^
		*Ze Xie Jia Wei decoction*	Guiding principles for clinical research on new drug of traditional Chinese Medicine.	^[36]^
		*Wenshenjianpihuatan Decoction*	Diagnosis and efficacy evaluation criteria of simple obesity	^[40]^
		*Wuling powder/ Wu Lin Decoction*	Endocrinology specialty diseases and Rheumatism Clinical diagnosis and treatment of Chinese medicine 2^nd^ Edi.	^[31,33]^
	Phlegm-dampness	*Fat and gas capsules*	Diagnosis and efficacy evaluation criteria of simple obesity	^[27]^
		*Sanziyangqin Decoction combined with Yueju Pill*	Internal Chinese medicine	^[34]^
Heat accumulation type	Stagnation of heat in Spleen and Stomach	*Xiere Huazhuo Decoction*	Internal Chinese medicine	^[29]^
	Stagnation of damp-heat in spleen and stomach	*Bangpungtongsung-san*	Guidelines for diagnosis and treatment of common diseases in internal Chinese medicine	^[35]^
	Stomach heat and dampness stagnation	*Jianfei Tiaozhi capsule*	Diagnosis and efficacy evaluation criteria of simple obesity	^[38]^
		*Peilianmahuangfang*	Guidelines for diagnosis and treatment of common diseases in internal Chinese medicine	^[39,41]^
	Damp-heat accumulation	*Qutan Qingwei Decoction*	Diagnosis and efficacy evaluation criteria of simple obesity	^[41]^
Liver-*qi* stagnation type	Spleen deficiency and Liver-qi stagnation	*JainPi ShuGan JiangZhi Formula*	Guiding principles for clinical research on new drug of traditional Chinese Medicine Diagnosis and efficacy evaluation criteria of simple obesity	^[28]^
	Spleen deficiency and stagnation of liver-qi and heat	*Daeshio Decoction*	Guiding principles for clinical research on new drug of traditional Chinese Medicine	^[37]^

The studies were assorted into 3 major types according to the pathology; the phlegm-dampness type, the heat accumulation type, and the liver-*qi* stagnation type. The phlegm-dampness type was counted in 8 RCTs, and it included only phlegm-dampness (n = 2) and phlegm-dampness with spleen deficiency (n = 6). The heat accumulation type was counted in 6 RCTs and commonly, it was related to the spleen and stomach. The type included stagnation of heat (or damp-heat) in the spleen and stomach, stomach heat and dampness stagnation, and damp-heat accumulation. The liver-*qi* stagnation type was collected in 2 RCTs and called a spleen deficiency and liver-*qi* stagnation or spleen deficiency and stagnation of liver-*qi* and heat.

‘Diagnosis and efficacy evaluation criteria of simple obesity’ was most frequently used in Seven RCTs as the diagnostic criteria for PI. ‘Internal Chinese medicine’, ‘Guiding principles for clinical research on the new drug of traditional Chinese medicine’, and ‘Guidelines for diagnosis and treatment of common diseases in internal Chinese medicine’ were all used in 3 RCTs, respectively. ‘Endocrinology specialty diseases and rheumatism-Clinical diagnosis and treatment of Chinese medicine, 2^nd^ Edi.’ was used in 2 RCTs. Three RCTs used 2 kinds of diagnostic criteria. “Guidelines for diagnosis and treatment of common diseases in internal Chinese medicine” and “Internal Chinese medicine” were used in 2 RCTs, and “Guiding principles for clinical research on the new drug of traditional Chinese medicine” and “Diagnosis and efficacy evaluation criteria of simple obesity” were used in 1 RCT.

### 3.4. Risk of bias

**Figure 2.**
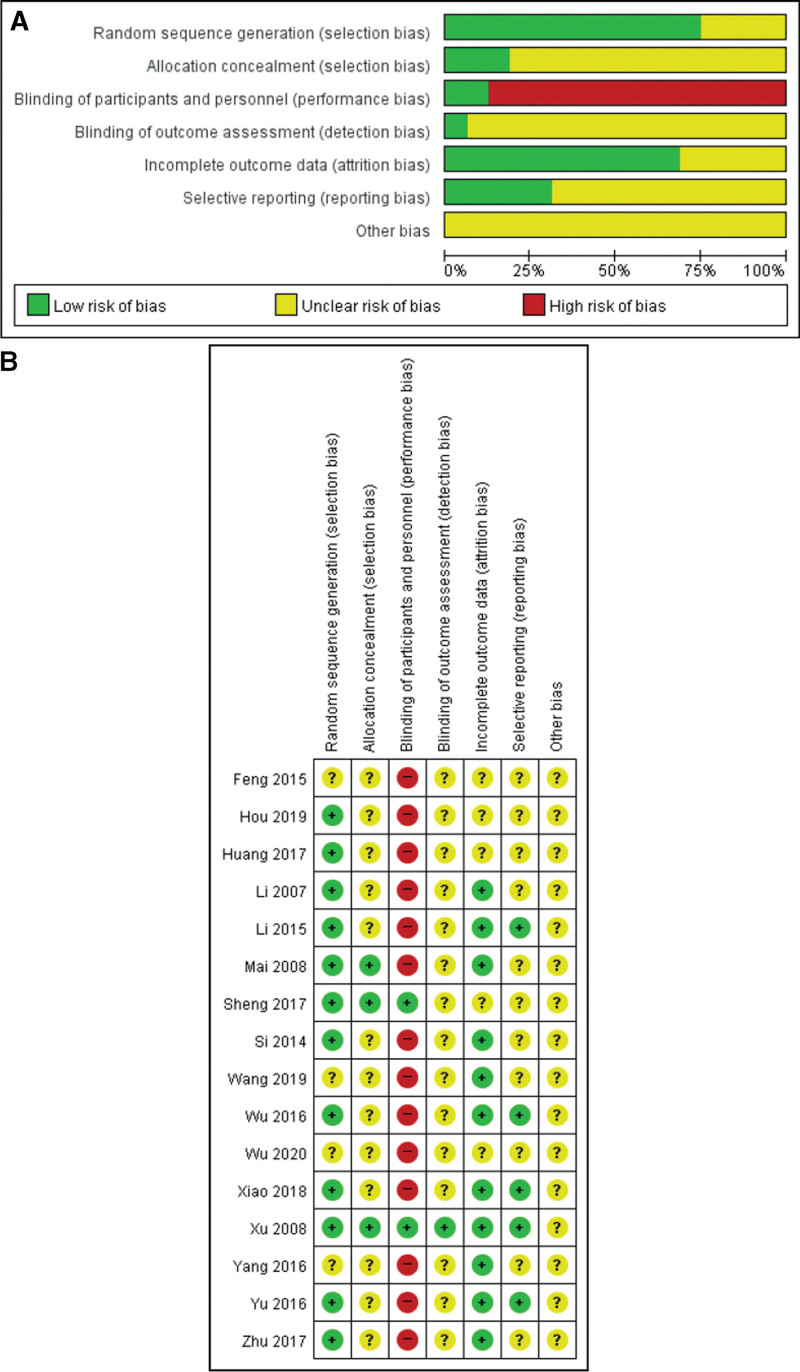
Methodological quality graph. (A) Risk of bias graph and (b) Risk of bias.

Fig. 2 indicates the risk of bias. In relation to selection bias, random sequence generation was low in 12 RCTs and unclear in 4. Allocation concealment was low in 3 RCTs and unclear in 13. Blinding of the participants and personnel was a high risk of bias in most studies, except for 2 RCTs that compared herbal medicine with a placebo. Another domain related to blinding and detection bias presented a low risk only in 1 RCT. Other RCTs did not describe sufficient information to assess the risk of bias. The risk of bias in incomplete outcome data was low in 11 RCTs and unclear in the others. The risk of selective reporting was assessed to be low in 5 RCTs; the risk of bias in the others was unclear, as there was a lack of explanation to assess the risk of bias. Most studies did not describe the information needed to assess the risk of bias; the other bias was also assessed as unclear. Only 2 RCTs whose comparator was placebo were assessed as having a low risk of bias overall. The others had a low or unclear risk of bias.

### 3.5. Outcomes

#### 3.5.1. BW and BMI.

Herbal medicine based on PI led to a significant reduction in both BW and BMI (BW: mean difference [MD] = –4.10, 95% confidence interval [CI]: –5.14 to –3.06, *P* < .0001, *I*^2^ = 2%, BMI: MD = –1.53, 95% CI: –1.88 to –1.19, *P* < .0001, *I*^2^ = 25%).

**Figure 3.**
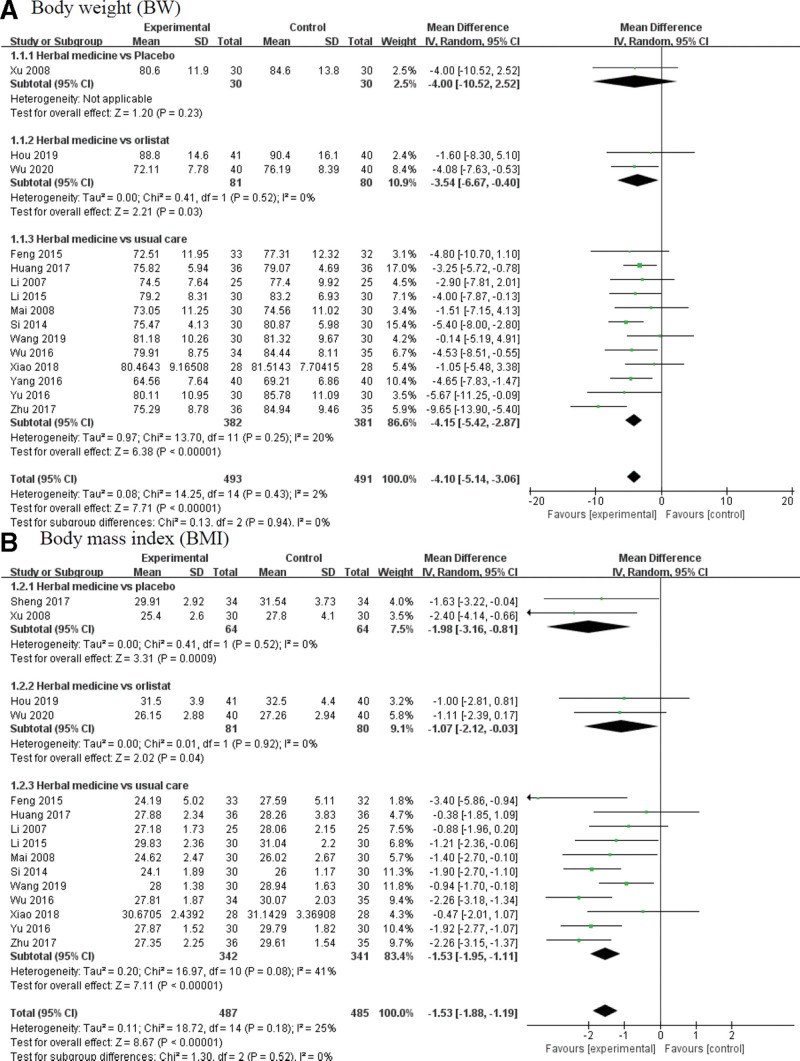
Forest plot of (A) body weight (BW) and (B) body mass index (BMI) for herbal medicine. BMI = body mass index, BW = body weight.

When herbal medicine was compared with comparators, the changes in BMI were statistically significant in all subgroup analyses. In subgroup analysis, however, which compared herbal medicine to placebo, the decrease in BW was not significant in the herbal medicine group compared to the placebo group (BW: MD = –4.00, 95% CI: –10.52 to 2.52, *P* = .23, *I*^2^ = not applicable). (Fig.3)

#### 3.5.2. WC, HC, and WHR.

The meta-analysis found that herbal medicine based on PI induced a significant decrease in WC, HC and WHR (WC: MD = –2.48, 95% CI: –2.95 to –2.02, *P* < .00001, *I*^2^ = 0%, HC: MD = –1.75, 95% CI: –3.21 to –0.29, *P* = .02; *I*^2^ = 65%, and WHR: MD = –0.03, 95% CI: –0.05 to –0.01, *P* = .0003; *I*^2^ = 80%).

**Figure 4.**
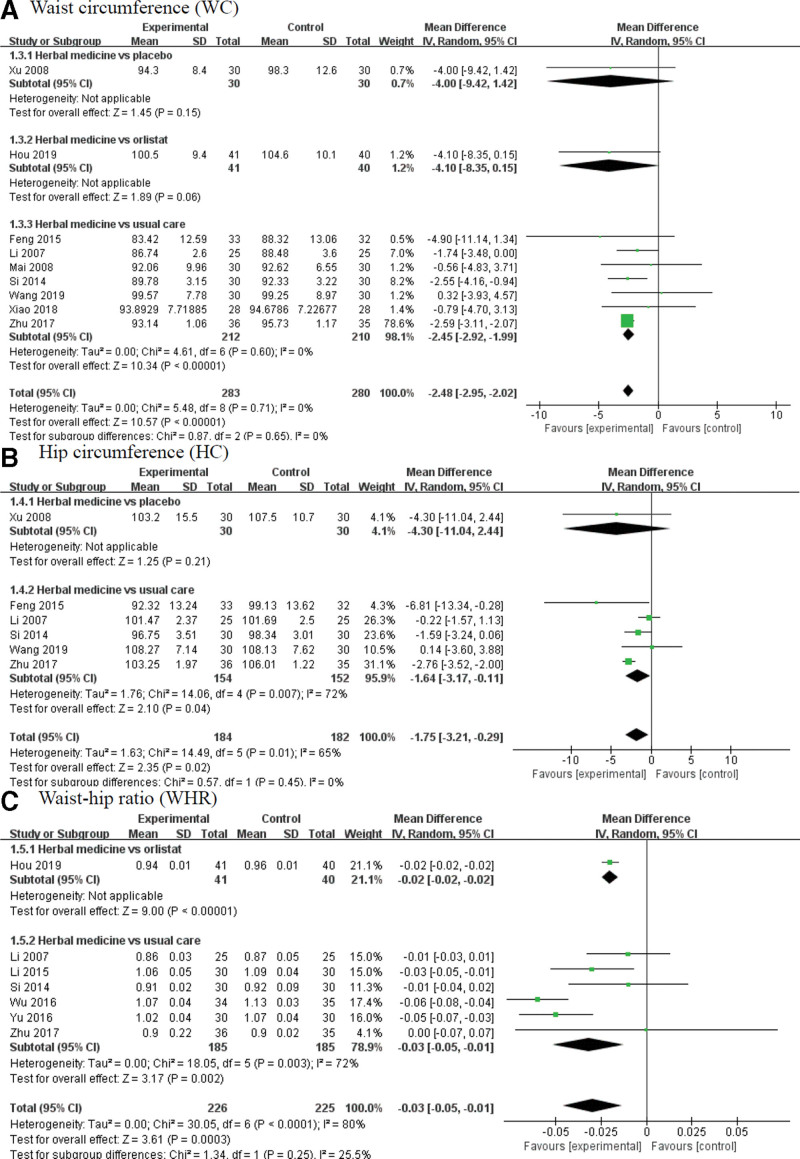
Forest plot of (A) waist circumference (WC); (C) hip circumference (HC), and (E) waist-hip ratio (WHR) for herbal medicine group compared to control group. HC = hip circumference.

After subgroup analysis according to comparator including placebo, orlistat, and usual care, herbal medicine induced a significant decrease only when compared with usual care. Herbal medicine could not lead to a significant improvement in WC and HC compared to the placebo. (Fig. 4)

### 3.6. Adverse events

Eight RCTs did not report AEs, whereas the other 8 RCTs reported the safety of herbal medicine. Only 2 studies reported AEs in the experimental group. On the other hand, they were mild AEs (4 cases), such as diarrhea, and they did not cause severe results.

### 3.7. Publication bias

**Figure 5.**
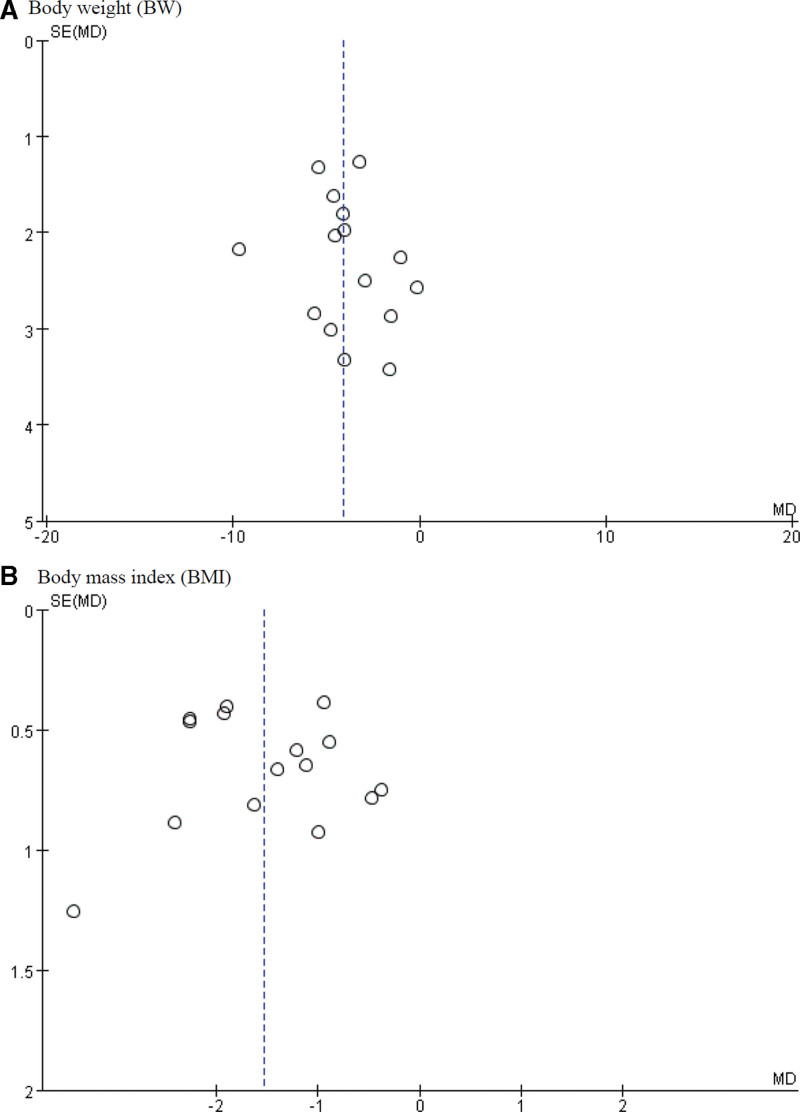
Funnel plots of (A) body weight (BW) and (B) body mass index (BMI) for herbal medicine compared to control group. The publication bias was only assessed when the number of studies included was over 10. BMI = body mass index, BW = body weight.

Fig.5 presents the publication bias according to the funnel plot. The funnel plot was considered visually asymmetric; hence, it was inferred that the publication bias possibly exists.

### 3.8. Assessment of evidence

The quality of evidence for the primary outcome, BW and BMI, was assessed as moderate with a high risk of blinding. The assessment of the evidence for WC was also presented moderate quality. On the other hand, the quality of evidence for HC and WHR was downgraded as “very low” and “low,” respectively, because of the serious inconsistency (high heterogeneity) and imprecision (small sample size).

## 4. Discussion

Obesity is an important disease because it is both weight gain and an indication of a health risk.^[1–3]^ In Korea, the adult obesity-related mortality rate is greater than 30%, and this increasing percentage is a cause of concern worldwide.^[43]^ In TCM, there are many attempts to find an effective treatment for obesity. Among those, the most frequently used treatment is herbal medicine, which is a prescription based on TCM theory.^[1,3]^ Although the most common treatment is this herbal medicine prescription based on PI, there are few reports examining the treatment effect and safety on obesity.

There are some previous reviews on the weight loss effects of herbal medicine; however, there is no systematic review and meta-analysis focusing on PI. Most studies researched only a single herb intervention or the mechanisms of herbal medicine.^[19–24]^ Some reported the efficacy of herbal medicine, but they were limited in 1 or several specific kinds of herbal formula.^[25]^ Some systematic reviews analyzed studies using herbal medicine without conducting meta-analysis, so there were limits to estimating the effect size of herbal medicine.^[26]^ There was another attempt to study herbal medicine prescriptions, but it was aimed to find out the current status of herbal medicine sales and investigate new anti-obesity medicine.^[44]^

In TCM, it is commonly understood that the prescription should differ according to the pathogenesis even if the diagnosis is the same. Therefore, it is important for effective treatment to determine the correct PI type and prescribe matched herbal medicine. There are 6 types in PI for obesity: phlegm, food retention, blood stasis, lever-*qi* stagnation, deficiency of *yang,* and deficiency of spleen. On the other hand, it could be largely divided into 3 types according to the results of this study. The phlegm-dampness type and the heat accumulation type were the most common, followed by liver-*qi* stagnation with a deficiency of spleen.

The common way to diagnose PI is through an examination of the symptoms in a subjective inspection. The phlegm-dampness type is characterized by feeling heavy, tired, headache, dizziness, bloating, or loose stool, which results from a dysfunction of the spleen.^[1,3]^ The deficiency of the spleen can induce a lack of circulating energy that leads to a lack of energy consumption and then obesity.^[1,3]^ On the other hand, the heat accumulation type is characterized by eating habits, frequent overeating or eating even after feeling full.^[1,3]^ Furthermore, constipation, stomach burning, thirst, or preferring cold water are common symptoms of this type, and it is similar to the food retention type.^[1,3]^ Overeating causes heat by stagnating food in the stomach and induces a dysfunction of the energy mechanism.^[1,3]^ The liver-*qi* stagnation type is related to stress.^[1,3]^ Chest tightness, chest fullness, upset, irritability, and irregular menstruation are common symptoms of the liver-*qi* stagnation type.^[1,3]^ It has been known that stress can cause a dysfunction of the digestive or systemic metabolism, such as deficiency of the spleen, endocrine disorder, and appetite abnormalities.^[1,2,4,5]^ The above 3 major types are assorted according to the characteristics of symptoms, and it is difficult to understand those 3 types as 1.

Considering the perspective of pathogenesis of obesity, it is valid that 3 PI types in the present result were the major types. In TCM, obesity is caused by several pathogenic problems, such as dampness, phlegm, and heat accumulation.^[1,3]^ Overeating of high-calorie and high-fat diets can result in a dysfunction of the stomach and spleen.^[1,3]^ The dysfunction of the organs can induce pathogenic dampness, phlegm, and heat, which are explained as pathogenic results of hyper- or hypo-production.^[1,3]^ If those are obstructed in the body, it is changed dampness with phlegm or occurs as heat accumulation that finally causes obesity.^[1,3]^

In previous studies conducted in Korea, the liver-*qi* stagnation type, phlegm type, food retention type, and deficiency of spleen type were the most commonly reported type of obesity.^[9,10,45,46]^ The results of the present study are similar to previous studies, but they are not the same. This was probably because there was a difference in the background in which the study was conducted. Most studies included in the present study were conducted in China. Therefore, there could be differences in the characteristics of subjects. In a previous report that analyzed studies on Chinese people, the deficiency of the spleen type with or without phlegm was reported the most, followed by the heat accumulation type and liver-*qi* stagnation type.^[47]^ Furthermore, there are differences in the diagnostic methods and PI terms. To increase the reliability, in Korea, the questionnaire, which is a point-based survey and unified 6 types developed by Korea institute of oriental medicine, is used widely as diagnostic methods.^[48–50]^ However, there is no unified diagnostic system and unified terms which are used widely in China. The present study showed that the main symptoms were similar, but the references of the diagnostic criteria for PI were different, even though it was the same PI type according to the pathogenic mechanism. Moreover, it was the same in the PI term; various terms indicated the same PI type. This can cause excessive subdivision of the PI system and the ratio of PI types to appear differently, as well as a misunderstanding of PI. Detailed analysis and classification are also important, but it is believed that utilization and reliability will increase if it is divided into major large categories and a unified method and terms are used.

In this study, the primary and additional outcomes were effectively improved. In all subgroup analyses, herbal medicine led to significant improvement in BMI, which is a major measurement of diagnosis of obesity. On the other hand, other measurements, including BW, WC, HC, and WHR, decreased insignificantly in the herbal medicine group compared to the placebo group. In this subgroup analysis, just 1 study was included, and it compared the anti-obese effectiveness between herbal medicine and a placebo after only 4 weeks.^[27]^ Considering that the study periods of the included articles were from 4 to 12 weeks, and most were conducted for 12 weeks, the period might be too short to induce a proper reduction of the anthropometric indices.

In relation to safety, half of the included studies reported AEs. After evaluating the functions of the liver and kidneys and examining other AEs, there were only 4 mild AEs in the experimental group.^[33,38]^ On the other hand, there was no significant difference between the experimental and control groups.^[33,38]^ Considering that the 2 studies compared herbal medicine with usual care without herbal medicine, there was no significant difference in safety. More AEs can occur when using herbal medicines unsuitable for PI because of different pathomechanisms.

Because of the difference in the ratio by PI type and ethical reason that herbal medicine that are not suitable for PI are at high risk of AEs, there was no study has conducted direct comparisons of the anti-obesity effects of herbal medicine depending on whether it was applied based on a PI or not. Most studies were designed to recruit suitable subjects using PI first and apply herbal medicine or control intervention later to participants diagnosed with the selected PI type.^[27–42]^ One study collected subjects without PI and diagnosed them with several PI types after recruitment.^[11]^ On the other hand, it also divided the subjects into 2 groups, applied herbal medicine or not, to determine the effectiveness of herbal medicine.^[11]^ Post-analysis was conducted to find out the differences according to whether the herbal medicine is suitable for PI or not, but the sample size was too small for reliable analysis.^[11]^

The effectiveness of specific herbal medicine without using PI and its effect size was reported in a previous study.^[25]^ The common herbal medicine, *BTS* and *TEJWT,* showed that both herbal medicines led to a decrease in BW and BMI but the changes were not statistically significant (*BTS* BW: MD = –0.32, 95% CI: –3.01 to 2.28, *P* = .82, *I*^2^ = 0% and BMI: MD = –0.67, 95% CI: –2.88 to 1.36, *P* = .48, *I*^2^ = 0%; *TEJWT* BW: MD = –2.38, 95% CI: –6.45 to 1.69, *P* = .25, *I*^2^ = 0% and BMI: MD = –0.46, 95% CI: –1.56 to 0.64, *P* = .41, *I*^2^ = 0%).^[25]^ On the other hand, in the present study, BW and BMI were reduced significantly in the herbal medicine group compared to the control group. The effect size of herbal medicine for BW and BMI was larger than the results of the previous study conducted without using PI.

Another study reported the effectiveness of *BTS* and *boiogito-tang(BGT*) and the tendency of AEs according to PI.^[11]^ *BTS* is representative herbal medicine for the heat accumulation type, and care should be taken when applying it to the deficiency type.^[1,3,11]^ *BGT* is a common herbal medicine for the deficiency type.^[1,3,11]^ Not far from these directions, it was revealed that *BTS* was more effective for the liver-*qi* stagnation type.^[11]^ On the other hand, when *BTS* was applied to the deficiency of *yang* type, there were more AEs, including dyspepsia, epigastric pain, diarrhea, and headache.^[11]^ Furthermore, more AEs were counted in the liver-*qi* stagnation type in the *BGT* group.^[11]^

The anti-obese effect and safety of herbal medicine cannot be compared directly using PI because of a lack of studies. Although it is not a study on obesity, there were studies that focused on whether herbal medicine according to PI is more effective or not. Studies on stroke reported that functional improvement in acute stroke patients depended on the correspondence between herbal medicine and PI. The score of functional recovery tended to improve further in correspondence group than non-correspondence group.^[51,52]^ In addition, the association between a specific herbal medicine and PI was also studied. *Gamichuongsangboha-tang*, which is used to treat asthma, is reported that its therapeutic effect lasts longer in patients with deficiency type compared to excess type.^[53]^ Another study reported the effectiveness of *Biyeom-go*, which is a herbal ointment for rhinitis, is associated with cold-heat pattern and that ointment can be more effective for patients with heat type compared to cold type.^[54]^ *Cheonwangbosim-dan*, that has been prescribed for insomnia patients, was studied and it improved sleep quality in insomnia patients with a heart-*yin* deficiency type compared to non-heat-*yin* deficiency type.^[55]^ It is hard to conclude that herbal medicine using PI is more effective for obesity, above studies can support the possibility of PI for further improvement on treatment of obesity.

Thus, this paper proposes the possibility that herbal medicine using PI is more effective than not using PI, considering that the anti-obese mechanisms of herbal medicine are not limited to reducing appetite and lipid absorption.^[23]^ The anti-obesity mechanism is explained by suppressing appetite, reducing the absorption of lipids and carbohydrates, inhibiting adipogenesis, regulating lipid and energy mechanisms, and improving the anti-obesity-related inflammation.^[23]^ Herbal medicine can be an excellent option not only to reduce body weight but also to solve the risk of health linked with obesity.^[20,23]^ PI reflects the holistic view of TCM, and it represents the patients’ symptoms. The TCM scores, which indicate the severity of the general symptoms related to the PI types, were decreased significantly with the weight reduction in most studies included. From these improvements, it is reasonable that herbal medicine regulates the weight changes and the systematic mechanism.

The notable point of this study Is the quality of evidence. The changes in BW, BMI, and WC were assessed as a “moderate” quality of evidence. On the other hand, the quality of evidence of HC and WHR was downgraded to “very low” and “low.” This resulted in a high risk of bias in blinding, high heterogeneity, and a smaller sample size than the optimal information size. The primary outcome should have moderate quality. Despite the high risk of performance bias caused by the study design, the results were reliable with a large enough sample size, low heterogeneity, and no serious publication bias or indirection.

Recently, there has been increasing interest in precision medicine, as with obesity research.^[56,57]^ PI can be an attractive option for individualized medicine, considering the developing procedures of precision medicine. For developing precision medicine, deep phenotyping of patients, such as medical history, lifestyle, physical examination, basic laboratory tests, imaging, functional diagnostics, and omics, is important.^[58]^ After preprocessing of these large data, data mining continues to establish diagnostic and prognostic models that leads to a prediction of the treatment response.^[58]^ Moreover, these tracts are fed back to the deep phenotyping stage.^[58]^ Because PI results from the historical accumulation of experience, deep phenotyping, which is essential to defining precision medicine, is prepared well. The next steps, including the setting of diagnostic and prognostic models and the predicting of treatment responses, have already been done using PI. The remaining work is a feed-back process for elaboration, which will require further studies using PI.

PI has been suggested attractive issue for many researchers because of its association with precision medicine.^[17,18,59]^ Using PI can contribute to increasing treatment efficiency and assessing patients state and treatment progress. The acute cerebral infarction patients with phlegm-dampness type showed higher score in functional recovery compared to patients with *Yin*-deficiency type.^[51]^ The treatment efficiency of tinnitus was higher in spleen-stomach weakness type, stomach heat type and phlegm-fire type than other types regardless of the aspect of tinnitus.^[60]^

Although PI has possibility of individual medicine, there are few issues to apply PI to research. Because PI diagnosis criteria are often based on subjective symptoms, limitation of standards for PI and model validity are hinder the expansion of studies using PI. To solve these problems, there were many attempts including proposing a suitable methodological solution or finding out biomarkers related to PI.^[61,62]^ Evaluation of PI questionnaire using data mining or machine learning has been conducted for improvement of validity.^[61]^ Biomarkers that have association with PI are studied in some diseases including coronary heart disease, rheumatoid arthritis, Gastric carcinoma, and other diseases.^[63–66]^ Those efforts make well designed further studies able and the utilization of PI increased.

This study had some limitations. First, pattern identification was not included in the search terms. Because it is not a controlled vocabulary, the search results decreased too much. Pattern identification was excluded from the search term to increase the sensitivity. Instead, all articles related to PI were extracted after the researcher screened the full text. On the other hand, there may be some studies that could not be included. Second, the included studies had a high risk of performance bias related to blinding, and those were performed in the same country. Hence, the results can be changed with further well-designed studies. Third, there was no direct comparison between herbal medicine with and without PI. Few studies reported the anti-obesity effect of herbal medicine according to the different PI groups; however, those had too few patients in each group, and there was no comparability among the groups. Next, all the included studies were conducted within 3 months, and it was difficult to evaluate the long-term effects of herbal medicine. Furthermore, the ignored heterogeneity according to the treatment period could be higher because the study designs focused on the short-term effects. Hence, a follow-up study will be needed. Lastly, the absence of unified PI diagnostic criteria and guidance of herbal medicine according to PI was a problem.

Despite the above limitations, this study is the first review to evaluate the efficacy and safety of herbal medicine focusing on PI in treating obesity. It is insufficient to clarify whether herbal medicine is more effective and safter than not using PI, this study is able to present a new perspective considering PI in the treatment of obesity. Further well-designed studies with large sample sizes will allow the results and quality of evidence to be upgraded. In addition, this paper proposed the possibility of PI as precision medicine, which can be a novel approach to obesity treatment. Nevertheless, more well-designed studies will be required to yield a high quality of evidence and clarify the effectiveness of herbal medicine based on PI.

In conclusion, 16 RCTs (2932 patients) focusing on herbal medicine with PI were reviewed, and the characteristics of PI used in the included studies were analyzed. The major 3 types of PI were the Phlegm-dampness type, heat accumulation type, and liver-qi stagnation type. The criteria for a diagnosis of PI presented 5 kinds of references. For anti-obese effectiveness, all outcome measurements were reduced significantly in the herbal medicine group compared to the control groups. The grade of evidence was of moderate quality, with a high risk of bias in the primary outcomes. Through analysis and assessment of its quality, herbal medicine is suggested to be an effective and safe treatment for obesity.

## Author contributions

**Conceptualization:** Dongho Keum, Hojun Kim.

**Data curation:** Seohyun Park.

**Formal analysis:** Seohyun Park.

**Funding acquisition:** Hojun Kim.

**Methodology:** Seohyun Park.

**Project administration:** Hojun Kim.

**Supervision:** Dongho Keum, Hojun Kim.

**Visualization:** Seohyun Park.

**Writing – original draft:** Seohyun Park.

**Writing – review & editing:** Hojun Kim.

## References

[1] The Society of Korean Medicine Rehabilitation. Korean Rehabilitation Medicine. 5th ed. Seoul: Kunja 2015. p. 299–319.

[2] World Health Organization. Obesity: preventing and managing the global epidemic: report of a WHO consultation. Geneva: World Health Organization 2000. 11234459

[3] The Korean Institute of Oriental Medicine. KMCPG (Korean Medicine Clinical Practice Guidelines)-Obesity. Seoul: Cheongun 2016.

[4] World Health Organization. Obesity and Overweight. World Health Organization. Available at: https://www.who.int/news-room/fact-sheets/detail/obesity-and-overweight. [access date June 21, 2022].

[5] Semlitsch T Stigler FL Jeitler K. Management of overweight and obesity in primary care – a systematic overview of international evidence-based guidelines. Obes Rev. 2019;20:1218–30. 31286668 10.1111/obr.12889 PMC6852048

[6] Nam GE Kim YH Han KD. Obesity fact sheet in Korea, 2020: prevalence of obesity by obesity class from 2009 to 2018. J Obes Metab Syndr. 2021;30:141–8. 34158420 10.7570/jomes21056 PMC8277583

[7] Cho YJ Lee AR Hwang MJ. Relationship between oriental obesity pattern, life habitual factors and psychological factors in Korean obese and overweight women. J Korean Med Obes Res. 2011;11:15–24.

[8] Chung WS Hwang MJ Lee AR. The difference of syndrome differentiation patterns between premenopausal and climacteric obese Korean women. J Korean Med Obes Res. 2008;8:37–47.

[9] Hwang MJ Park JS Song MY. Analysis of oriental obesity pattern identification questionnaire on overweight and obese Korean adult women. J Korean Med Obes Res. 2008;8:63–72.

[10] Kim EJ Lee AR Hwang MJ. Relationship between visceral adipose tissue and oriental obesity pattern identification in obese Korean women. J Korean Med Rehabil. 2011;21:279–88.

[11] Park JS Song YK Hwang EH. Analysis of development and application of pattern identification system -based on oriental obesity pattern identification. J Korean Med Rehabil. 2014;24:107–14.

[12] Park JH Kim HJ. Clinical practice recommendations for *bangpungtongseong-san* (*Bofutsusho-san*) and *bangkihwangki-tang* (*Boiogito*) in obesity. J Korean Med Obes Res. 2012;12:48–58.

[13] Kim SH Jeong JH Lim JH. Acupuncture using pattern-identification for treatment of insomnia disorder: a systematic review and meta-analysis of randomized controlled trials. Integr Med Res. 2019;8:216–26. 31497504 10.1016/j.imr.2019.08.002 PMC6718809

[14] Ang L Lee HW Choi JY. Herbal medicine and pattern identification for treating COVID-19: a rapid review of guidelines. Integr Med Res. 2020;9:100407. 32289016 10.1016/j.imr.2020.100407 PMC7104236

[15] Jiao Y Liu J Jiang L. Guidelines on common cold for traditional Chinese medicine based on pattern differentiation. J Tradit Chin Med. 2013;33:417–22. 24187858 10.1016/S0254-6272(13)60141-7 PMC7148786

[16] Birch S. Treating the patient not the symptoms: acupuncture to improve overall health – evidence, acceptance and strategies. Integr Med Res. 2019;8:33–41. 30949430 10.1016/j.imr.2018.07.005 PMC6428918

[17] Mei MF. A systematic analysis of the theory and practice of syndrome differentiation. Chin J Integr Med. 2011;17:803–10. 22057408 10.1007/s11655-011-0890-0

[18] Jiang M Lu C Zhang C. Syndrome differentiation in modern research of Chinese medicine. J Ethnopharmacol. 2012;140:634–42. 22322251 10.1016/j.jep.2012.01.033

[19] Payab M Hasani-Ranjbar S Shagbal N. Effect of the herbal medicines in obesity and metabolic syndrome: a systematic review and meta-analysis of clinical trials. Phytother Res. 2020;34:526–45. 31793087 10.1002/ptr.6547

[20] Shin SS Yoon M. Regulation of obesity by antiangiogenic herbal medicines. Molecules. 2020;25:4549. 33020443 10.3390/molecules25194549 PMC7582783

[21] Hasani-Ranjbar S Nayebi N Larijiani B. A systematic review of the efficacy and safety of herbal medicines used in the treatment of obesity. World J Gastroentero. 2009;15:3073–85. 10.3748/wjg.15.3073 PMC2705729 19575486

[22] Heber D. Herbal preparations for obesity: are they useful?. Primary Care. 2003;30:441–63. 14567158 10.1016/s0095-4543(03)00015-0

[23] Shang A Gan RY Xu XY. Effects and mechanisms of edible and medicinal plants on obesity: an updated review. Crit Rev Food Sci Nutr. 2021;61:2061–77. 32462901 10.1080/10408398.2020.1769548

[24] Jo GW Ok JM Kim SY. Review on the efficacy and safety of mahuang and ephedrine in the treatment of obesity -focused on RCT. J Korean Med. 2017;38:170–84.

[25] Han K Lee MJ Kim H. Systematic review on herbal treatment for obesity in adults. J Korean Med Rehabil. 2016;26:23–35.

[26] Park JH Lee MJ Song MY. Efficacy and safety of mixed oriental herbal medicines for treating human obesity: a systematic review of randomized clinical trials. J Med Food. 2012;15:589–97. 22612295 10.1089/jmf.2011.1982

[27] Xu H. The clinical research of conditioning triple energizer on simple obesity [master’s thesis]. Guangzhou: Guangzhou University 2008.

[28] Sheng Z Hu Y Liu J. Effect of JianPi ShuGan JiangZhi formula on simple obesity and the expression of leptin and adiponectin. World Chin Med. 2017;12:587–90.

[29] Hou R Liu Q Jin X. Clinical analysis of Xiere Huazhuo decoction in treatment of obesity. (Stagnation of Heat in Spleen and Stomach). Liaoning J TCM. 2019;46:65–8.

[30] Wu H Zhou K. Clinical study on modified Liujunzi Tang combined with orlistat for simple obesity with syndrome of spleen deficiency with dampness obstruction. J New Chin Med. 2020;52:43–6.

[31] Feng S He C Li W. Observation of BMI and blood lipid changes of simple obesity of spleen deficiency and phlegm damp type by Wuling powder. Chin Med Pharmacol. 2015;5:67–9.

[32] Huang W Pan F Huang J. Clinical research on reformatted Linggui Zhugan decoction in the treatment of simple obesity with spleen deficiency dampness obstruction syndrome. J Yangtze Univ. 2017;14:4–6.

[33] Mai X. Clinical observation on Wu Lin decoction to curing the simple obesity [master’s thesis]. Guangzhou: Guangzhou University 2008.

[34] Wang J. Clinical study of Sanziyangqin decoction and Yueju pill on obesity of phlegm-dampness type [MSD thesis]. Taiyuan: Shanxi University 2019.

[35] Wu W. Clinical observation of the efficacy of Fangfengtongsheng-wan cure the pure obesity patients and its impacts on LP and APN [master’s thesis]. Harbin: Heilongjiang University 2016.

[36] Xiao T. A clinical study on the effect of Ze Xie Jia Wei decoction on the treatment of spleen-induced obesity and its effect on body fat rate [master’s thesis]. Fuzhou: Fujian University 2018.

[37] Zhu Y. Clinical observation of modified Daeshio decoction in treating simple obesity of liver stagnation and spleen deficiency and heat syndrome. J Clin Med Lit. 2017;4:679–80.

[38] Li S Zai J Wang Y. A clinical study on treatment of simple obesity (Stomach-heat and damp-stagnation syndrome) by Jianfei Tiaozhi capsule. J New Chin Med. 2007;39:28–9.

[39] Li J. Observe the simple obesity treatment efficacy of the Peilianmahuang-fang and research on its effects on the INS, GLP-1 and IAPP [master’s thesis]. Harbin: Heilongjiang University 2015.

[40] Si Y Xiang N. Clinical observation of Wenshenjianpihuatan decoction in treatment of simply obesity. Hubei J TCM. 2014;35:11–2.

[41] Yang Y. Clinical observation of “Qutan Qingwei Decoction” in treating simple obesity of damp-heat accumulation. Shanghai J TCM. 2016;50:62–3.

[42] Yu H. Observe the efficacy of Pei Lian Ma Huang Fang in the treatment of simple obesity and the impact of LPS [master’s thesis]. Harbin: Heilongjiang University 2016.

[43] Committee of Clinical Practice Guidelines, Korean Society for the Study of Obesity (KSSO). 2018 Korean Society for the Study of Obesity Guideline for the management of obesity in Korea. J Obes Metab Syndr. 2019;28:40–5. 31089578 10.7570/jomes.2019.28.1.40 PMC6484940

[44] Lee MS. A study on oriental medicine prescription for the effect of obesity treatment [master’s thesis]. Gwangju: Chosun University 2019.

[45] Song MY Kim HJ Lee MJ. Relation between obesity pattern identification and metabolic parameters in overweight and obese women. J Korean Med Obes Res. 2014;14:24–8.

[46] Yoo JE Cho YH Gu HG. Relation between metabolic syndrome and obesity pattern identification questionnaire in middle-aged health check-up examinees. J Korean Med. 2014;35:124–34.

[47] Gao Y Wang Y Zhou J. Effectiveness of electroacupuncture for simple obesity: a systematic review and meta analysis of randomized controlled trials. Evid Based Compl Alternat Med. 2020;2020:2367610. 10.1155/2020/2367610 PMC7341404 32714399

[48] Kang BG Moon JS Choi SM. A reliability analysis of syndrome differentiation questionnaire for obesity. Korean J Orient Med. 2007;13:109–14.

[49] Kang KW Moon JS Kang BG. The discrimination model for the pattern identification diagnosis of overweight patients. Korean J Orient Med. 2008;14:41–6.

[50] Kang KW Moon JS Kang BG. The comparison of pattern identification diagnosis according to symptom scale based on obesity pattern identification questionnaire. J Korean Med Obes Res. 2009;9:37–44.

[51] Lee EC Park SK Kwak SH. Differences of symptom improvement depending on correspondence of herb medicine with oriental medical diagnosis in acute stroke patient. Korean J Joongpoong. 2011;12:8–15.

[52] Hyun SH. Analysis of changes in clinical indicators and function improvement and the effect of herbal prescriptions in accordance to pattern identification in acute cerebral infarction pateints [MSD thesis]. Seoul: Kyunghee University 2015. 10.1155/2015/517158 PMC4615209 26523149

[53] Choi JY Lee JS Jeong SY. An analysis of therapeutic effects of *Gamichuongsangboha-tang* in 30 asthmatics based on criteria for deficiency-excess differentiating syndromes of asthma. Korean J Orient Int Med. 2004;25:379–87.

[54] Son MJ Lee DH. Effectiveness of herbal ointment *Biyeom-go* according to cold-heat pattern identification: a subgroup analysis on patients with rhinitis. J Korean Med Ophthalmol Otolaryngol Dermatol. 2019;32:29–40.

[55] Kim MJ Bose S Shin NR. The herbal formula CWBSD improves sleep quality dependent on oral microbial type and tongue diagnostic features in insomnia. J Pers Med. 2021;11:325. 33919176 10.3390/jpm11050325 PMC8143156

[56] Lee MS. Research trends in obesity & obesogenic environments in Korea. Nutr Res Pract. 2019;13:461–72. 31814921 10.4162/nrp.2019.13.6.461 PMC6883237

[57] Cifuentes L Maria DHA Jeanette EP. Precision medicine for obesity. Dig Dis Interv. 2021;5:239–48. 36203650 10.1055/s-0041-1729945 PMC9534386

[58] Konig IR Fuchs O Hansen G. What is precision medicine?. Eur Respir J. 2017;50:1700391. 29051268 10.1183/13993003.00391-2017

[59] Birch S Alraek T Bovey M. Overview on pattern identification-history, nature and strategies for treating patients: a narrative review. Eur. J. Integr. Med. 2020;35:101101.

[60] Kim GJ. Analysis of tinnitus pattern by visceral pattern identification and treatment efficiency by pattern identification type. J Korean Med Ophthalmol Otolaryngol Dermatol. 2019;32:77–86.

[61] Oh JH Wang JH Choi SM. Re-evaluation of obesity syndrome differentiation questionnaire based on real-world survey data using data mining. J Korean Med Obes Res. 2021;21:80–94.

[62] Chung VCH Ho RST Wu X. Incorporating traditional Chinese medicine syndrome differentiation in randomized trials: methodological issues. Eur J Integr Med. 2016;8:898–904.

[63] Zhou H Li L Zhao H. A large-scale, multi-center urine biomarkers identification of coronary hear disease in TCM syndrome differentiation. J Proteome Res. 2019;18:1994–2003. 30907085 10.1021/acs.jproteome.8b00799

[64] Wu G Zhao J Zhao J. Exploring biological basis of syndrome differentiation in coronary heart disease patients with two distinct syndromes by integrated multi-omics and network pharmacology strategy. Chin Med. 2021;16:109. 34702323 10.1186/s13020-021-00521-3 PMC8549214

[65] Seca S Franconi G. Understanding Chinese medicine patterns of rheumatoid arthritis and related biomarkers. Medicines(Basel). 2018;5:17. 29401671 10.3390/medicines5010017 PMC5874582

[66] Sun DZ Xu L Wei PK. Syndrome differentiation in traditional Chinese medicine and E-cadherin/ICAM-1 gene protein expression in gastric carcinoma. World J Gastroenterol. 2007;13:4321–7. 17708604 10.3748/wjg.v13.i32.4321 PMC4250857

